# Effects of reductions in US foreign assistance on HIV, tuberculosis, family planning, and maternal and child health: a modelling study

**DOI:** 10.1016/S2214-109X(25)00281-5

**Published:** 2025-09-17

**Authors:** John Stover, Emily Sonneveldt, Yvonne Tam, Katherine C Horton, Andrew N Phillips, Jennifer Smith, Rowan Martin-Hughes, Debra ten Brink, Daniel T Citron, Hae-Young Kim, Adam Akullian, Edinah Mudimu, Michael Pickles, Anna Bershteyn, Jessica Williamson, Gesine Meyer-Rath, Lise Jamieson, Elizabeth A Sully, Julia N White, Alexis Heaton, Rebecca A Clark, Hannah Tong, Alexandra S Richards, C Finn McQuaid, Rein M G J Houben, Richard G White, Dobromir Dimitrov, David Kaftan

**Affiliations:** a Avenir Health, Glastonbury, CT USA; b Department of International Health, Johns Hopkins Bloomberg School of Public Health, Baltimore, MD, USA; c Department of Infectious Disease Epidemiology and Dynamics, London School of Hygiene and Tropical Medicine, London, UK; d Department of Infection and Population Health, University College London, London, UK; e Burnet Institute, Melbourne, VIC, Australia; f Department of Population Health, New York University Grossman School of Medicine, New York, NY, USA; g School of Public Health, Imperial College London, London, UK; h Health Economics and Epidemiology Office (HE2RO), Faculty of Health Sciences, University of the Witwatersrand, Johannesburg, South Africa; i The South African Department of Science and Innovation/National Research Foundation Centre of Excellence in Epidemiological Modelling and Analysis (SACEMA), Stellenbosch University, Stellenbosch, South Africa; j Guttmacher Institute, New York City, NY, USA; k Reproductive Health Supplies Coalition, Washington, DC, USA; l Institute for Disease Modeling, Gates Foundation, Seattle, Washington, USA; m Department of Decision Sciences, University of South Africa, Pretoria, South Africa; n Vaccine and Infectious Diseases Division, Fred Hutchinson Cancer Center, Seattle, WA, USA

## Abstract

**Background:**

The USA has traditionally been the largest donor to health programmes in low-income and middle-income countries (LMICs). In January 2025, almost all such funding was stopped and prospects for its resumption are uncertain. The suddenness of the funding cuts makes it difficult for national health programmes in LMICs to adapt. We aimed to estimate the impact of these cuts on deaths and other outcomes (new infections, number of family planning users, and unplanned pregnancies) for four health areas that have been a focus of a substantial amount of US foreign assistance: HIV, tuberculosis, family planning, and maternal and child health.

**Methods:**

We applied established mathematical models to the countries receiving US foreign assistance in each domain to estimate health impacts over the period 2025 to 2030. We used six models of HIV, three different approaches to estimate family planning impact, and one model each for tuberculosis and maternal and child health, applying these models to as many as 80 countries. We compared model projections assuming constant funding (status quo) with projections assuming complete elimination of US funding in each country. Some models also considered partial cuts or restoration of funding over time.

**Findings:**

A complete cessation of US funding without replacement by other sources would lead to drastic increases in deaths from 2025 to 2030: 4·1 million (range 1·6–6·6) additional AIDS-related deaths across 55 countries, 606 900 (95% uncertainty interval [UI] 466 000–768 800) additional tuberculosis deaths across 79 countries, 40–55 million additional unplanned pregnancies and 12–16 million unsafe abortions across 51 countries, and 2·5 million (1·3–4·5) additional child deaths from causes other than HIV and tuberculosis across 24 countries. Restoration of funding for HIV treatment but not prevention would avoid most of the increase in deaths but still result in nearly 1 million more new HIV infections from 2025 to 2030.

**Interpretation:**

Substantial progress has been made in improving global health in the past few decades. This progress has strengthened hope in reaching global development goals. However, the recent funding cuts threaten to change these trajectories and could lead to sharp increases in avoidable mortality for the poorest countries. Even a partial restoration of US funding would combat the most severe effects and provide time for countries that have received substantial US foreign assistance to adjust to the new funding landscape.

**Funding:**

Economic and Social Research Council; Engineering and Physical Sciences Research Council; European and Developing Countries Clinical Trials Partnership; Gates Foundation; Global Fund to Fight AIDS, Tuberculosis, and Malaria; Open Philanthropy; UK Foreign, Commonwealth & Development Office; UK Medical Research Council; UN Population Fund; UNAIDS; US National Institute of Allergy and Infectious Diseases; University of Edinburgh; US National Institutes of Health; US President's Emergency Plan for AIDS Relief; Wellcome Trust; World Bank; WHO.

## Introduction

The USA has been a world leader in providing foreign assistance to enhance health, economic development, and security. Health has constituted a small part (12%) of this assistance. Historically, the USA has provided more funding for health programmes in low-income and middle-income countries (LMICs) than any other donor country. Over the past 5 years, the USA has provided US$12–13 billion annually (excluding the special appropriation for COVID-19 in 2021),^1^ accounting for about 25% of all donor assistance for health worldwide.^2^ These funds have supported programmes addressing HIV, global health security, maternal and child health, malaria, family planning, tuberculosis, and other conditions. US support accounts for 40% of donor funding for family planning^3^ (13% of all family planning funding in LMICs), 50% of donor funding for tuberculosis^4^ (8% of all tuberculosis expenditures in LMICs), and 73% of donor funding for HIV^5^ (27% of all HIV expenditures in LMICs). These funds have contributed to unprecedented gains in global health, including reductions in HIV infections and deaths (39% and 51%, respectively, from 2010 to 2023),^6^ reductions in child deaths (59% reduction in mortality in children <5 years [hereafter referred to as under-5 mortality]) from 1990 to 2022),^7^ increases in the number of couples using modern family planning methods (25% increase from 2012 to 2024),^8^ and reductions in tuberculosis deaths (23% reduction from 2015 to 2023).^4^

**Research in context**

**Evidence before this study**

On June 4, 2025, we searched PubMed for articles published since Jan 20, 2025, with the terms “PEPFAR” , “USAID”, or “funding” in the title. We found numerous policy pieces and editorials but only ten studies that tried to quantify the impact of the recent US funding cuts on health programmes related to HIV, tuberculosis, family planning, and maternal and child health. Cluver and colleagues estimated 0·5 million additional AIDS deaths in children by 2025; Tram and colleagues used a model of HIV in South Africa to estimate a 36% increase in AIDS deaths in South Africa in 2025; Kimmel and colleagues used a model of HIV in Rwanda to project 29 000–64 000 additional AIDS deaths over the next 10 years; Hontelez and colleagues used the STDSIM model to estimate 60 000–74 000 additional AIDS deaths in seven low-income and middle-income countries (LMICs) due to a 90-day funding freeze; ten Brink and colleagues applied the Optima model to 26 LMICs to estimate 0·77–2·93 million additional AIDS deaths by 2030 due to cuts in international assistance from the US President's Emergency Plan for AIDS Relief (PEPFAR) and other international donors. Nichols and colleagues developed websites to estimate the number of additional AIDS deaths and tuberculosis deaths that have occurred since the freeze started. Mandal and colleagues estimated an additional 99 800 tuberculosis deaths in 26 LMICs as a result of US funding cuts. Clark and colleagues found that termination of US assistance could lead to 420 000 additional tuberculosis deaths by 2025 in 79 LMICs. Menzies and colleagues estimated that the withdrawal of US assistance could lead to 340 000 additional paediatric tuberculosis deaths up to 2034 in 130 countries.

**Added value of this study**

This is, to the best of our knowledge, the first in-depth modelling study of the effects of the US funding cuts on HIV, tuberculosis, family planning, and maternal and child health that includes all countries receiving US funding. Well established models were applied to all countries previously receiving US support for these programmes.

**Implications of all the available evidence**

We found that the cuts to US funding, if made permanent, would reverse decades of progress in global health and lead to large increases in deaths and new cases of HIV and tuberculosis that would return to the levels of the 1990s. The USA is likely to restore some funding for HIV treatment but not prevention, while prospects for restored funding for tuberculosis, family planning, and maternal and child health are slim. Some upper-middle-income countries will increase domestic funding for some programmes to replace the lost funds, but for the poorest countries, where US aid constitutes a third to half of domestic funding for health and education, the scope to replace US development assistance is insufficient. Our estimates should be updated once the funding situation and domestic governments’ responses become clear.

A substantial reduction or elimination of US funding for global health could therefore have drastic consequences for the people most reliant on this assistance, leading to many additional deaths, unplanned pregnancies, abortions, and disabilities. Previous research has estimated impact in some locations for HIV^9^, ^10^, ^11^, ^12^, ^13^, ^14^ and tuberculosis,^15^, ^16^, ^17^ but not for all affected countries. We aimed to estimate the increase in HIV, tuberculosis, and maternal and child deaths as well as unplanned pregnancies in individual countries and globally as a result of the recent cuts in US foreign assistance.

## Methods

### Overview

For each health area we modelled a status quo scenario with 2024 levels of funding continuing until 2030, leading to constant coverage of all interventions, and a “no US funding” scenario with all US funding removed, causing a decline in coverage for those interventions supported by US funding. For example, in Zambia in 2024, US funding supported 53% of antiretroviral therapy (ART) for people living with HIV and 10% of family planning commodities. A complete cessation of US funding would cause ART coverage to drop to from 94% to 44% and contraceptive prevalence to drop from 37% to 33%.

For HIV we also modelled a partial resumption of funding for treatment in line with the most recent budget proposals. For the other sectors, especially family planning, it is not clear at present whether there will be any future funding. All modelling was done at the country level and aggregated to estimate global impact. Table 1 shows the countries included in each health domain and the percentage of funding provided by the USA.

**Table 1:** Percentage of total funding provided by the USA

	**HIV: total funding from PEPFAR**	**Tuberculosis: national tuberculosis programme budget from US funding**	**Family planning: modern family planning users using USAID-funded commodities in 2024**	**Maternal and child health**				
				Antenatal and postnatal care supported by US funding	Skilled birth attendance and emergency obstetric care supported by US funding	Essential newborn care supported by US funding	Small and sick newborn care supported by US funding	Diagnosis and treatment of childhood illnesses supported by US funding
Afghanistan	..	37%	1%	19%	19%	10%	10%	10%
Angola	22%	10%	7%	..	..	..	..	..
Azerbaijan	..	5%	..	..	..	..	..	..
Bangladesh	..	38%	3%	19%	19%	10%	10%	10%
Benin	84%	0%	16%	..	..	..	..	..
Bolivia	..	27%	..	..	..	..	..	..
Botswana	45%	..	..	..	..	..	..	..
Brazil	..	18%	..	..	..	..	..	..
Burkina Faso	31%	31%	51%	..	..	..	..	..
Burundi	67%	10%	..	..	..	..	..	..
Cambodia	23%	35%	..	..	..	..	..	..
Cameroon	88%	..	1%	..	..	..	..	..
Chad	..	33%	..	..	..	..	..	..
China	..	32%	..	..	..	..	..	..
Colombia	4%	10%	..	..	..	..	..	..
Congo (Brazzaville)		56%	..	..	..	..	..	..
Côte d'Ivoire	60%	..	21%					
Dominican Republic	40%	10%	..	..	..	..	..	..
DR Congo	48%	..	25%	23%	32%	19%	27%	27%
El Salvador	14%	6%	..	..	..	..	..	..
Equatorial Guinea	..	37%	..	..	..	..	..	..
Eritrea	..	1%	..	..	..	..	..	..
Eswatini	58%	47%	5%					
Ethiopia	64%	38%	1%	19%	19%	10%	10%	10%
Fiji	..	47%	..	..	..	..	..	..
Gabon	..	7%	..	..	..	..	..	..
The Gambia	..	35%	..	..	..	..	..	..
Ghana	12%	11%	28%	3%	5%	3%	7%	7%
Guatemala	19%	8%	..	..	..	..	..	..
Guinea	..	31%	..	..	..	..	..	..
Haiti	92%	..	49%	19%	19%	10%	10%	10%
Honduras	35%	..	..	..	..	..	..	..
India	8%	7%	..	..	..	..	..	..
Indonesia	12%	32%	..	..	..	..	..	..
Iraq	..	2%	..	..	..	..	..	..
Jamaica	49%	..	..	..	..	..	..	..
Jordan	..	11%	..	..	..	..	..	..
Kazakhstan	4%	2%	..	..	..	..	..	..
Kenya	41%	..	12%	3%	5%	3%	7%	7%
Kyrgyzstan	24%	27%	..	..	..	..	..	..
Laos	72%	24%	..	..	..	..	..	..
Lesotho	57%	11%	3%	..	..	..	..	..
Liberia	72%	46%	6%	19%	19%	10%	10%	10%
Madagascar	..	38%	21%	23%	32%	19%	27%	27%
Malawi	49%	34%	29%	19%	19%	10%	10%	10%
Mali	23%	..	60%	19%	19%	10%	10%	10%
Mauritania	..	38%	..	..	..	..	..	..
Moldova	..	8%	..	..	..	..	..	..
Mongolia	..	8%	..	..	..	..	..	..
Mozambique	69%	51%	87%	19%	19%	10%	10%	10%
Myanmar	79%	41%	..	..	..	..	..	..
Namibia	51%	4%	5%	..	..	..	..	..
Nepal	90%	14%	..	..	..	..	..	..
Nicaragua	40%	..	..	..	..	..	..	..
Niger	..	30%	7%					
Nigeria	74%	44%	1%	3%	5%	3%	7%	7%
Pakistan	32%	..	..	..	..	..	..	..
Panama	21%	..	..	..	..	..	..	..
Papua New Guinea	12%	24%	..	..	..	..	..	..
Paraguay	..	1%	..	..	..	..	..	..
Peru	9%	2%	..	..	..	..	..	..
Philippines	11%	23%	..	..	..	..	..	..
Rwanda	59%		41%	19%	19%	10%	10%	10%
Senegal	35%	14%	16%	19%	19%	10%	10%	10%
Sierra Leone	26%	..	..	..	..	..	..	..
South Africa	18%	11%	..	..	..	..	..	..
South Sudan	47%	..	..	35%	42%	27%	49%	42%
Sri Lanka	..	4%	..	..	..	..	..	..
Sudan	..	36%	..	..	..	..	..	..
Suriname	..	7%	..	..	..	..	..	..
Syria	..	38%	..	..	..	..	..	..
Tajikistan	24%	13%	..	..	..	..	..	..
Tanzania	76%	..	33%	19%	19%	10%	10%	10%
Thailand	3%	8%	..	..	..	..	..	..
Timor-Leste	..	38%	..	..	..	..	..	..
Uganda	65%	..	44%	19%	19%	10%	10%	10%
Ukraine	12%	27%	..	..	..	..	..	..
Vanuatu	..	34%	..	..	..	..	..	..
Venezuela	..	24%	..	..	..	..	..	..
Viet Nam	27%	38%	..	..	..	..	..	..
Yemen		38%	..	..	..	..	..	..
Zambia	84%	45%	16%	19%	19%	10%	10%	10%
Zimbabwe	56%	72%	9%	..	..	..	..	..

PEPFAR=US President's Emergency Plan for AIDS Relief.

The main outcome was the number of additional deaths in the “no US funding” scenario compared to the “status quo” scenario. We also included additional HIV infections, family planning users, unplanned pregnancies, and abortions.

Table 2 provides information on each of the models used in this analysis.

**Table 2:** Characteristics of models

	**Institution**	**Health area**	**Model type**	**Number of countries included**
Goals	Avenir Health	HIV	Compartment infectious disease model	55
Optima	Burnet Institute	HIV	Compartment infectious disease model	13
EMOD	Institute for Disease Modeling	HIV	Agent-based infectious disease model	6
Synthesis	University College London	HIV	Agent-based infectious disease model	2 (Malawi, Zimbabwe)
PopART	Imperial College London	HIV	Agent-based infectious disease model	1 (Zimbabwe)
Thembisa	University of Cape Town	HIV	Compartment infectious disease model	1 (South Africa)
LSHTM	London School of Hygiene & Tropical Medicine	Tuberculosis	Compartmental model	79
Impact2	MSI Reproductive Choices	Family planning	Commodity-based analysis	41
LiST	Johns Hopkins University	Maternal and child survival	Dynamic cause of death model	25

USAID=US Agency for International Development.

### HIV/AIDS

We estimated the impact of the US funding cuts on AIDS deaths and new HIV infections for the 55 countries previously supported by the US President's Emergency Plan for AIDS Relief (PEPFAR). Six different well established HIV models were used, and the results were averaged at the country level before aggregating to a global total. All models were calibrated to surveillance, survey, and programme data on HIV prevalence and AIDS mortality available for each country. The Goals model^18^ was applied to all 55 countries and calibrated to trends in the UNAIDS-supported official Spectrum/AIM estimates; the Optima HIV model^19^ was applied to 13 countries, accounting for 33% of deaths in all PEPFAR countries in 2024; the HIV Synthesis model^20^ was applied to Malawi and Zimbabwe; the PopART-IBM model^21^ was applied to Zimbabwe; the Thembisa model^22^ was applied to South Africa; and the EMOD-HIV model^23^, ^24^, ^25^ was applied to Eswatini, Kenya, Malawi, South Africa, Zambia, and Zimbabwe, accounting for 27% of AIDS-related deaths in all PEPFAR countries in 2024. Uncertainty ranges represent the country-weighted average difference between the lowest and highest estimates across all countries with multiple modelled estimates. A summary of the structure, simulation methods, and calibration methods for each model is available from the HIV Modelling Consortium.

We used data on PEPFAR expenditures for HIV as a proportion of total expenditures from recipient country resource alignment reports^26^ and UNAIDS National HIV Spending Assessments. We did not include PEPFAR contributions to the Global Fund to Fight AIDS, Tuberculosis, and Malaria due to the difficulty of assigning these contributions to specific countries and the uncertainty about the future of US contributions to the Global Fund. We assumed that the coverage of HIV testing, treatment, and prevention services would drop from 2024 levels to a lower level in 2025–30, proportional to the share of all HIV funding provided by PEPFAR in each country for treatment and prevention separately. Across all 55 countries, PEPFAR accounted for 40% of all HIV expenditures in 2022–23, ranging from less than 5% to 92%. By US law, 70% of PEPFAR's overall funding is for treatment and palliative care, 20% is for prevention, and 10% is for orphans and vulnerable children, although we used country-specific distributions that might differ from this pattern. Modelling included the effects of funding cuts on testing, treatment, prevention of mother-to-child transmission, condom availability, voluntary medical male circumcision, pre-exposure prophylaxis; and services for sex workers, men who have sex with men, and people who inject drugs. We also considered the effects of continuing funding for HIV treatment but not prevention.

### Tuberculosis

We used a dynamic, compartmental model of *Mycobacterium tuberculosis* transmission, progression, and care^27^ to simulate tuberculosis epidemic trajectories. The model structure is shown in the appendix (p 1).

For each country, we modelled a scenario reflecting complete cessation of funding to national tuberculosis programmes from the US Agency for International Development (USAID) and from US contributions to the Global Fund. We did not model indirect impacts from reductions in funding to health systems, HIV programmes, or international agencies that provide technical assistance to countries. WHO budget data^28^ were used to calculate the proportion of total expected funding for all budget line items each country expected from USAID and from the Global Fund in 2023. We combined these proportions with estimated US contributions via the Global Fund using data on pledged donations for the Seventh Replenishment (2023–25).^29^

Funding cuts are likely to have a direct impact on tuberculosis treatment by restricting the accessibility of tuberculosis services and the availability of tuberculosis diagnostics and treatment. Thus, we assumed a reduction in treatment initiation rates proportional to budget reductions. We assumed that funding cuts will be sustained from 2025 into the future and estimated cumulative excess incident episodes of symptomatic tuberculosis and tuberculosis-associated deaths through to 2030.

Of 111 LMICs with data available to attempt calibration, complete budget data were available for 90 countries. Model calibrations were completed for 79 of 90 countries, representing 91% of global tuberculosis incidence and 90% of global tuberculosis mortality in 2023.^4^

### Family planning

Family planning impact estimates are provided by three different organisations. The Guttmacher Institute and the Reproductive Health Supplies Coalition (RHSC) provide insight into the short-term loss of USAID funding in 2025, with Guttmacher focusing on the impact of total funding and RHSC providing insight into the funding gaps for procurement of contraceptive commodities in 24 donor-supported countries with available data. Avenir Health estimated the impact of just the loss in contraceptive commodities for the next 5 years, from 2025 to 2030.

Guttmacher Institute estimates include US foreign assistance for family planning (at the country level) from congressional appropriations^3^, ^30^ to estimate the number of family planning users by using country-specific estimates of cost per user. This approach accounts for funding not only for commodity and direct service delivery but also USAID investments into the larger programmes and systems costs that support family planning within a country. Impacts were estimated with the Adding it Up methodology, which estimates pregnancy rates and outcomes assuming non-use of contraceptives. The RHSC estimates are based on data from the Global Family Planning Visibility and Analytics Network (VAN). This analysis combines known procurement commitments with estimates of procurement needs as per government supply plans or as per scenarios developed with reported inventory and consumption levels. The output is the required funding to maintain the desired stock levels set by each country from March 2025 through to December 2025. Commodity costs are increased by 20% to account for freight cost, highlighting the additional costs needed to get commodities into countries.

Avenir Health used data from the RHSC's Reproductive Health Supplies Visualizer (RH Viz), which combines historical procurement data with live procurer shipment data from the VAN. The data on condoms were adjusted to remove 74% of the products supplied to HIV programmes.^31^ Data on the total volume of products procured across 34 countries were entered into the Impact2 model^32^ to estimate the total the number of full-time equivalent users (couple-years of protection) that could be supported by these commodities and to estimate the impacts of the elimination of USAID commodity funding. Commodity procurement data for 2024 were not yet fully available at the time of writing, so the impacts presented are a range of data between 2023 and 2024. Annual procurement volumes are held constant to 2030. For long-acting methods (eg, implants and intrauterine devices [IUDs]) the full lifetime impacts are included. It is assumed that all commodities are used and that USAID funding is not replaced by others.

### Maternal and child health

Impact estimates for maternal and child health and stillbirths were projected with the Lives Saved Tool (LiST).^33^ LiST estimated changes in cause-specific mortality for those populations that were previously supported by USAID in 25 countries. Funding supported activities to strengthen these health systems and improve the supply chain, which then improves health service delivery and health intervention coverage.

We established the baseline health statuses in 2024 with country-specific mortality rates, causes of death, and coverage of interventions. Three scenarios were modelled to illustrate the change in death counts if coverage of interventions decreased without recovery or was maintained at current levels. To estimate the reduction in coverage of interventions, countries were categorised by the amount of official development assistance funding as a percentage of their global health expenditure as less than 5% (no impact, five countries), 5–9% (small impact, three countries), 10–29% (moderate impact, 14 countries), 30–50% (large impact, two countries), and more than 50% (extreme impact, one country). The percentage reduction in workforce, supplies, and access to health services, which affects health intervention coverage, was then estimated by the five groups of countries and by packages of interventions of antenatal and postnatal care, skilled birth attendants and emergency obstetric care, essential newborn care, small and sick newborn care, prevention of childhood illnesses (including vaccines), and diagnosis and treatment of childhood illnesses. In total, reductions in coverage were applied to 48 interventions, listed in the appendix (p 3).

### Role of the funding source

The funders of the study had no role in study design, data collection, data analysis, data interpretation, or writing the report.

## Results

Across all four sectors, our analysis estimates that there could be as many as 8 million additional deaths from 2024 to 2030 (appendix p 4). Details by sector are provided below. Overlap between tuberculosis and HIV deaths and competing risks across all sectors mean that the actual total would be somewhat less than the sum of the individual sector results.

**Figure 1:**
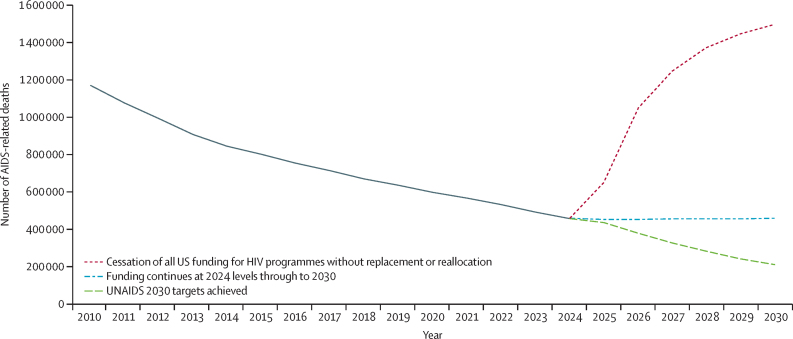
Effect of US funding cuts on AIDS-related deaths in 55 PEPFAR-supported countries by scenario Results are the sum across all countries of the average results for all models applied to each country. PEPFAR=US President's Emergency Plan for AIDS Relief.

A complete cessation of US funding, resulting in a permanent loss of US-funded HIV/AIDS programmes, could result in 4·1 million (range 1·6–6·6) additional AIDS-related deaths from 2025 to 2030 across 55 countries compared with continued funding at 2024 levels; the range represents the variation in results across models that were applied in the same countries. Mozambique, Nigeria, South Africa, Tanzania, Uganda, and Zambia would account for 2·3 million of these additional deaths. The annual number of AIDS-related deaths could increase from 470 000 (as per AIDSinfo) in 2023 to 1·5 million by 2030, far above the 510 000 deaths expected if current programmes continued and eight times higher than the UNAIDS 2030 target of a 90% reduction from 2010 (implying no more than 230 000 deaths in 2030 in all of these countries; figure 1). The historical decline in deaths was due largely to increasing treatment coverage. If this coverage were to remain constant, then the number of deaths would stop declining.

During the same period there could be 7·5 million (range 4·7–10·2) additional new HIV infections, which would lead to even more deaths beyond 2030 and increases in the number of people living with HIV, from 33 million in 2023 to 36 million by 2030. The impacts would be particularly hard on children, with 350 000 (range 270 000–440 000) additional child deaths due to AIDS and 690 000 (420 000–950 000) additional HIV infections. If current trends were to continue, the number of children orphaned by AIDS would drop from 11·4 million in 2024 to 7·9 million by 2030, but with cuts to US assistance the number of orphans due to AIDS would be roughly constant at 11·3 million in 2030.

Continuing funding for HIV treatment but not prevention could reduce the worst projected impacts substantially. Relative to the full cuts scenario, across countries where models estimated a partial resumption scenario, 99% of additional deaths could be averted by maintaining treatment. However, relative to status quo, these cuts to HIV prevention and other services (representing up to 20% of US funding) could result in nearly 1 million additional new HIV infections from 2025 to 2030. Results for the six HIV models when applied to the same countries vary somewhat due to model structure, although all models are calibrated to the same HIV prevalence data.

**Figure 2:**
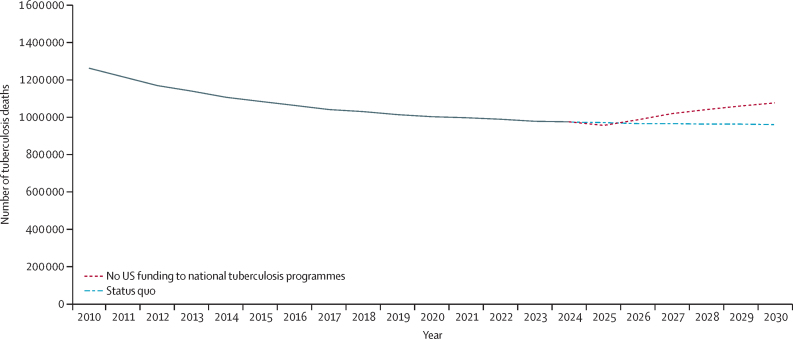
Impact of cuts to US funding on global tuberculosis deaths in 79 low-income and middle-income countries

Results show that a loss of all US funding to national tuberculosis programmes, without replacement, could result in 606 900 (95% uncertainty interval [UI] 466 000–768 800, based on model calibrations) additional tuberculosis deaths across 79 countries between 2025 and 2030, a 10·3% (7·7–12·2) increase compared to the status quo (figure 2). Over this same period, this loss of funding would result in an additional 16·1 million (95% UI 14·3–18·0) new *M tuberculosis* infections, representing a 9·5% (7·0–11·8) increase, and 1·2 million (0·9–1·5) incident episodes of symptomatic tuberculosis, representing a 2·0% (1·4–2·8) increase.

US Government appropriations for family planning assistance globally have been steady, at approximately $600 million annually for the past 9 years, as outlined in the US foreign assistance budget dataset. This funding from the US Government includes programmatic support, systems strengthening, service delivery, and procurement of products. The Guttmacher Institute estimates that withholding the full appropriated funding in 2025 is estimated to result in 17·1 million more unintended pregnancies, including 7·6 million unplanned births and 5·2 million unsafe abortions, which will in turn result in 34 000 more maternal deaths per year.^34^

For 2025, RHSC estimates the current contraceptive procurement funding gap in 32 countries at $167 million ($200 million assuming 20% for freight costs). If the pending USAID procurement commitments do not materialise this year, then the funding gap for these countries could increase by 26%, to $43 million ($51 million assuming 20% for freight costs). The potential $43 million added gap represents 70% of the $61 million worth of family planning products the US Government has historically funded and is in line with the trend from previous years at this point in the year, given procurement seasonality.

**Figure 3:**
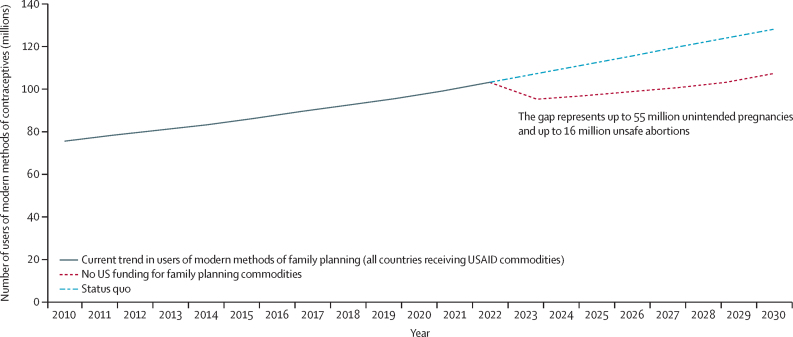
Effect of US funding cuts on the number of people using modern methods of family planning in 34 countries The 34 countries are Afghanistan, Angola, Benin, Bangladesh, Burkina Faso, Côte d’Ivoire, Cameroon, DR Congo, Eswatini, Ethiopia, Ghana, Haiti, Jordan, Kazakhstan, Kenya, Lesotho, Liberia, Madagascar, Malawi, Mali, Mozambique, Namibia, Nepal, Niger, Nigeria, Rwanda, Senegal, Sierra Leone, Tanzania, Togo, Uganda, Ukraine, Zambia, Zimbabwe. USAID=US Agency for International Development.

In 2023 alone, USAID purchased 106 million male condoms for family planning and reproductive health, 26·5 million cycles of oral contraceptives, 23·4 million doses of injectable contraceptives, 2·8 million contraceptive implants, 300 000 IUDs, and almost 500 000 doses of emergency contraception. The totality of contraceptives funded by USAID and shipped in 2023 represented almost 18 million person-years of use (couple-years of protection). USAID's sustained investment in delivering contraceptives from 2025 to 2030 could have supported 73–100 million users of contraceptives across 41 countries over this period (figure 3). This loss could result in an additional 40–55 million unintended pregnancies among women across 51 countries, with the largest estimated impacts in Tanzania, DR Congo, Uganda, and Mozambique (the range is based on annual fluctuations in USAID commodities provided). These additional unintended pregnancies could result in 15–20 million abortions across 51 countries, more than 75% of which we estimate would be unsafe (12–16 million). Since most programmes maintain stocks equal to 3–6 months of consumption and some previous purchases might still be in transit or at ports for custom clearance, the full impacts could be delayed until 2026.

**Figure 4:**
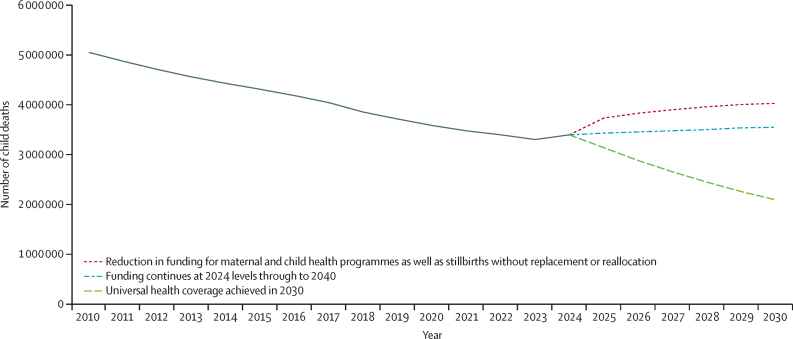
Impact of US funding cuts on child deaths in the 25 countries with USAID support The 25 countries are Afghanistan, Bangladesh, Congo (Brazzaville), Ethiopia, Ghana, Haiti, India, Indonesia, Kenya, Liberia, Madagascar, Malawi, Mali, Mozambique, Myanmar, Nepal, Nigeria, Pakistan, Rwanda, Senegal, South Sudan, Tanzania, Uganda, Yemen, and Zambia. USAID=US Agency for International Development.

Cuts to US development assistance would reverse the long trend of decline in maternal and child deaths and stillbirths. Due to aid cuts, the assumed average absolute reduction in coverage is 14% (range 7–26) for antenatal and postnatal care; 15% (8–28) for skilled birth attendants and emergency obstetric care; 9% (4–16) for essential newborn care; 9% (5–18) for small and sick newborn care; 11% (5–19) for prevention of childhood illnesses (including vaccines); and 10% (5–19) for diagnosis and treatment of childhood illnesses. Results from the LiST model show that the maternal mortality ratio would increase by 18·4% (range 13·0–27·0), the under-5 mortality rate would increase by 16·4% (9·7–27·1), and the stillbirth rate would increase by 9·0% (5·6–13·6) compared with 2024 values. From 2025 to 2030 there would be an additional 2·5 million (1·3–4·5) child deaths, compared to the status quo scenario, across 24 countries. By 2030, child deaths would be almost double the number expected if universal health coverage could be achieved (figure 4); ranges are based on the assumed range of reduction in coverage. By 2030, the projected number of child deaths would be comparable to the levels in 2017. From 2025 to 2030, there would be 140 000 (range 79 000–236 000) additional maternal deaths due to a shortage of health-care services, plus (from the family planning calculations described above) at least an additional 200 000 deaths that could result from increased unintended pregnancies and exposure to complications during pregnancy and childbirths due to the loss of family planning services. There would also be 634 000 (range 348 000–1 033 000) additional stillbirths in the same period, compared to the status quo scenario.

## Discussion

Our analysis suggests that the impact of the US funding cuts would be enormous and would reverse the substantial progress made in HIV, tuberculosis, family planning, and maternal and child health over the past 15 years. There could be up to 8 million additional deaths from 2024 to 2030 across just these four sectors. Children would be especially hard hit, with 2·5 million additional deaths and more than 11 million orphans due to AIDS by 2030.

There is considerable uncertainty in these estimates due to several factors. We assumed that the coverage of all supported services would decline in direct proportion to US funding. In reality, national programmes will try to reallocate available resources to maintain the most critical services. Some countries have the capacity to increase domestic funding, although the most affected countries are also the poorest, with the least capacity to increase domestic funding or divert funds from other domestic priorities. Other donors could help to fill some of the gap, but already France, Germany, the Netherlands, Switzerland, and the UK have signalled their intention to cut their own contributions. The compounding effects this would have on top of US funding cuts have already been estimated for HIV.^13^ By contrast, some impacts are underestimated; for example, we did not model the intersection of HIV care and tuberculosis, for which reductions in ART access risk re-escalating tuberculosis in many countries in sub-Saharan Africa. Additionally, there are many cases where the USA only funds part of a programme (eg, just commodities or just service delivery) but the loss of that critical component could lead to the failure of the entire programme. There are also cases where the USA supports a large percentage of the overall system, such as logistics for medical commodities and drugs. In these cases, the impacts would be more extensive if the system either partially or completely collapsed. The USA has also provided substantial funding for technical assistance and guideline development through WHO, GAVI, the Vaccine Alliance, and other organisations; its withdrawal from these processes is not modelled here. In many areas, US funding has transformed the way data are collected and analysed, such as support for electronic medical record systems for ART patients and electronic logistics management systems for supply chain management. Without this support we would have much less data to guide patient care, programme strategy, and evaluation of progress.

There will be additional impact from cuts to malaria programmes since the USA contributes about 37% of all malaria funding in LMICs, so the impact is likely to be substantial. Modelling of these effects will be published separately.

In addition to the effects on mortality that are the focus of the current study, there will be effects on equity since these cuts are likely to focus most heavily on public services for the poorest segments of the population.

Our use of separate models for each area could have led to some double counting of deaths, particularly for HIV and tuberculosis, but the overlap with childhood mortality is small. In fact, synergistic effects would probably exacerbate the situation. For example, when funding for community health workers supporting people with HIV is lost then many of these individuals will go to health clinics, further constraining the ability of these clinics to provide all other health services.

For the maternal and child health analysis, reductions in coverage of interventions were only applied to interventions with publicly available coverage estimates, which could underestimate our estimated impact since there are proven interventions whose coverage is not measured or reported. For the maternal and child health analysis, baseline coverages of proven interventions in the Essential Newborn Care and Small and Sick Newborn Care packages were estimated from readiness-adjusted utilisation of health facility delivery, as coverages of those interventions were not measured or reported. The proxy coverages might either overestimate or underestimate the actual intervention coverages, which could lead to corresponding overestimation or underestimation of the impact. In several cases, we modelled intermediate funding scenarios in addition to the complete cessation of US development assistance in order to gauge the impact if the USA restores some funding. At any level of intermediate funding, optimisation could limit the negative impacts. These and other models are used by country programmes to seek the best use of available funds. However, donors, particularly the USA, often include restrictions on how their funding can be used that might not always allow for maximum impact.

All the models used for this analysis are well established in their fields and have been thoroughly reviewed by expert advisory groups and through peer review of publications. Nevertheless, there is some variation in results, as seen in the countries where multiple HIV models have been applied. However, the variation is generally small compared to the magnitude of the impacts. We also did not conduct a sensitivity analysis to assess which data sources and model assumptions had the greatest effects on outcomes. These analyses have been conducted for the individual models in previous studies and were beyond the scope of the multidisease, multimodel analysis provided here.

Our focus was on the effects of the cuts and the benefits of restoring US funding. Several policy papers have been published recently that provide recommendations on what to do if funding is not restored, such as the one by Singh and colleagues.^34^

Without substantial investment from alternative sources, a complete cessation of US funding would reverse decades of progress. Many countries, including many in sub-Saharan Africa, have been making efforts to increase domestic funding of health programmes, many through expansion of universal health-care programmes. However, these transitions cannot take place overnight.

Given the time needed to increase domestic funding in a sustainable manner, much of the dependence on donor assistance could gradually be eliminated. National programmes will find ways to compensate for some of the negative effects by increasing domestic funding, mobilising other resources, re-allocating funding, and optimising the way in which services are delivered. However, an abrupt, permanent end to US funding will not provide the time needed to adjust. The result is likely to be a large increase in avoidable deaths and new cases that portend a worsening situation in the years ahead.

### Contributors

### Data sharing

All models were calibrated with publicly available datasets as described in the article and references for each model. Also, as described in the current study, several of the models used (Goals, Optima, and EMOD) are available for free download from their respective websites. Procurement funding gap data from the Global Family Planning Visibility and Analytics Network (VAN) is subject to the VAN Terms of Use in terms of data access and sharing. VAN follows transparent data-sharing processes aligned with the Gates Foundation and other RHSC donor policies. Calibrations and projections of specific models for specific countries are available by request to the authors.

## Declaration of interests

We declare no competing interests.
