# Supplementary material for: Effects of reductions in US foreign assistance on HIV, tuberculosis, family planning, and maternal and child health: a modelling study
Source: Lancet Glob Health. 2025 Sep 17;13(10):e1669–80. doi: 10.1016/S2214-109X(25)00281-5 (PMC12447089; doi:10.1016/S2214-109X(25)00281-5)
Supplement: Supplementary appendix [file mmc1.pdf]

THE LANCET
Global Health

Supplementary appendix

This appendix formed part of the original submission and has been peer reviewed. We post it as supplied by the authors.

Supplement to: Stover J, Sonneveldt E, Tam Y, et al. Effects of reductions in US foreign assistance on HIV, tuberculosis, family planning, and maternal and child health: a modelling study. *Lancet Glob Health* 2025; **13:** e1669–80.

The Effects of Reductions in United States Foreign Assistance on HIV, TB, Family Planning and Maternal and Child Health: A Modeling Study

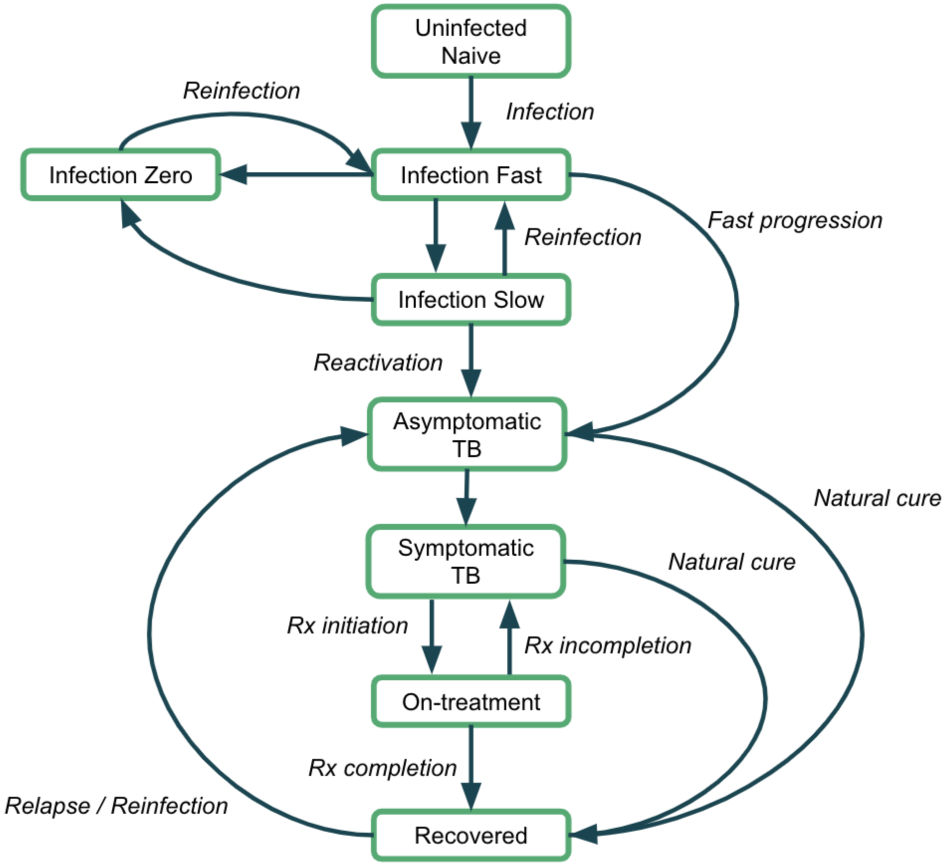

Supplemental Figure 1. TB Model structure. For each country, we calibrated the model to epidemiological data using history matching with emulation through the hmer package in R[20], generating 200 fitted parameter sets per country. Each country model was independently fitted to nine calibration targets in 2023: the country-specific TB incidence rate (for all ages, those aged 0–14 years, and those 15 years and older, separately), country-specific TB notification rate (for all ages, those aged 0–14 years, and those 15 years and older, separately), country-specific TB mortality rate (for all ages), and the global fraction of asymptomatic TB among infectious TB (asymptomatic + symptomatic). Models for countries classified as having a high TB burden due to HIV were fit to at least three

additional country-specific all-age targets in 2019: HIV prevalence, ART coverage, TB incidence rate in people living with HIV, and TB mortality rate in people living with HIV. We used the distribution of results produced by these parameter sets to quantify estimation uncertainty.[21]

Supplemental Table 1. Description of LiST MCH scenarios

**Scenario 1: Extreme impact (e.g. Afghanistan)**	**Workforce reduction**	**Supply reduction**	**Access**
**ANC, PNC**	Large	Extreme	Large
**SBA + Emergency obstetric care (basic and comprehensive)**	Large	Extreme	Extreme
**Essential Newborn Care**	Large	Large	Large
**Small and Sick Newborn Care**	Large	Extreme	Large
**Preventing childhood illness and death**	Extreme	Extreme	Extreme
**Diagnosis and treatment of childhood illness**	Extreme	Extreme	Large
**Rationale**	Programs that depend more heavily on CHWs will be more impacted by ODA reductions.	Programs that depend on specialized supplies and equipment will be most impacted (medications, IV fluids, vaccines, antimalarials, diagnostic kits). Provision of essential newborn care is primarily dependent on equipment availability.	Interventions implemented at the facility will be most impacted. Closure of facilities, increases out-of-pocket expenses and loss of cash support programs/incentives for patients (travel allowance, food vouchers) will decrease accessibility.
**Scenario 2: Large impact (e.g. Rwanda)**	**Workforce reduction**	**Supply reduction**	**Access**
**ANC, PNC**	Moderate	Large	Large
**SBA + Emergency obstetric care (basic and comprehensive)**	Moderate	Large	Extreme
**Essential Newborn Care**	Moderate	Moderate	Large
**Small and Sick Newborn Care**	Moderate	Large	Large
**Preventing childhood illness and death**	Large	Large	Large
**Diagnosis and treatment of childhood illness**	Large	Large	Large

Supplemental Table 2. Global Coverage by Model

Model	Health Area	Number of Countries Included	Number of deaths in included countries	Percent increase in deaths in included countries
**Goals**	HIV	55	490,000	150%
**Optima**	HIV	13	180,000	180%
**EMOD**	HIV	6	121,000	80%
**Synthesis**	HIV	2 (Malawi, Zimbabwe)	30,000	460%
**PopART**	HIV	1 (Zimbabwe)	19,000	390%
**Thembisa**	HIV	1 (South Africa)	50,000	44%
**LSHTM**	TB	79	970,000	6%
**LiST**	Maternal and child survival	25	7.3 million	195%
**Impact2**	Family planning	41		40-55 million unplanned pregnancies
